# Influence of conditional cash transfer program on prenatal care and nutrition during pregnancy: NISAMI cohort study

**DOI:** 10.1590/1516-3180.2021.0449.R1.23112021

**Published:** 2022-08-08

**Authors:** Jerusa da Mota Santana, Marcos Pereira, Cinthia Soares Lisboa, Djanilson Barbosa Santos, Ana Marlucia Oliveira

**Affiliations:** I MSc, PhD. Adjunct Professor, Health Sciences Center, Universidade Federal do Recôncavo da Bahia (UFRB), Cruz das Almas (BA), Brazil.; II MSc, PhD. Adjunct Professor, Instituto de Saúde Coletiva, Universidade Federal da Bahia (UFBA), Salvador (BA), Brazil.; III MSc. Doctoral Student, Universidade Estadual de Feira de Santana (UEFS), Feira de Santana (BA), Brazil.; IV MSc, PhD. Adjunct Professor, Health Sciences Center, Universidade Federal do Recôncavo da Bahia (UFRB), Cruz das Almas (BA), Brazil.; V MSc, PhD. Full Professor, School of Nutrition, Universidade Federal da Bahia (UFBA), Salvador (BA), Brazil.

**Keywords:** Feeding behavior, Public policy, Pregnancy, Prenatal care, Dietary patterns, Social protection, Family health

## Abstract

**BACKGROUND:**

There are few studies on the influence of a cash transfer program on nutritional outcomes from pregnancy.

**OBJECTIVES:**

To analyze how a Brazilian conditional cash transfer program (Bolsa Familia Program, BFP) was associated with changes in body mass index (BMI) and food consumption among pregnant women.

**DESIGN AND SETTING:**

Cohort study on 250 pregnant women (≥ 18 years of age) in Brazilian prenatal services.

**METHODS:**

A food frequency questionnaire was used to evaluate dietary intake. Weight was measured in each gestational trimester. Generalized estimation equations and structural equation modeling were used for statistical analyses. Correlations were analyzed using standardized coefficients (SCs).

**RESULTS:**

Women benefitting from the BFP were of greater age and had lower education. The BFP exerted a direct negative effect on the pregnant women’s consumption choices regarding refined grains, regional foods, vegetable oil, sausages, salted meats and snacks (SC = -0.10) and on maternal BMI (SC = -0.12). Among the intermediate variables, we observed that the time elapsed since pregnancy and the month of prenatal onset had direct negative effects; and that the number of visits to doctors, family income and number of years of education had direct positive effects.

**CONCLUSIONS:**

Beneficiaries were less likely to increase their BMI outside of the recommended standards and had a greater tendency to receive prenatal care. Participation in the BFP had a direct negative effect on adherence to unhealthy diets.

## INTRODUCTION

Social protection programs for health have grown in popularity around the world, especially for pregnant women within a context of vulnerability to hunger and food insecurity.^1–5^ These programs have important effects with regard to improving maternal and child health and nutrition and diminishing child and maternal mortality rates.^6^ However, there is an important gap in knowledge regarding interventions through such programs on the diets and gestational weight gains of women living in low and middle-income countries. Thus, epidemiological studies on the repercussions of social protection policies on the health and nutrition of pregnant women are important for evaluating these intervention programs.

There is evidence to suggest that social protection policies such as the Brazilian conditional cash transfer program (Bolsa Familia Program, BFP) attenuate the effects of poverty and ensure the human right to adequate food, thereby promoting food and nutritional security (FNS).^7,8^

In order to remain in the BFP, families must comply with requirements within the areas of education and health. With regard to health, these requirements include prenatal monitoring for pregnant beneficiaries, attendance at scheduled visits to doctors and educational activities relating to breastfeeding and adequate and healthy feeding during pregnancy and early childhood.^9^

The results from some studies have shown that pregnant women in the BFP begin prenatal care earlier and have a greater number of visits to doctors^6,10^ than those who are not program beneficiaries. The results indicate that the BFP constitutes a protective factor for the health of the mother-child binomial. This is due to early attention to improvement of health, thus resulting in adequate and healthy nutrition; and to adoption of preventive measures with regard to risk factors that compromise proper development of gestation, especially in the initial cycles of fetal formation.

In addition, beneficiaries of this program have made progress in seeking out nutritional care. In 2012, approximately 165,000 Brazilian pregnant women were monitored by healthcare teams. Among these women, 99% were up-to-date with their prenatal care and 80% had undergone evaluation of their nutritional status.^11^

Regarding the destination of the funds provided by the BFP, the results show that most families used the benefit to purchase foodstuffs. This proportion was higher in the northeastern region and among families in situations of greater food and nutritional insecurity (FNI).^12,13^

An epidemiological survey conducted in Brazil revealed changes to the population’s diet after its integration into the BFP. This contributed to greater food and nutritional security among these households through increasing their access to foods such as vegetables, eggs, oils, fruits, beans, meats, grains, milk, biscuits, processed foods and sugars.^14^ According to the beneficiaries, the food groups most consumed were rice and grains and milk, with a smaller percentage for vegetables and roots.^14^

In relation to the maternal-infant group, studies have shown that the BFP had positive effects on maternal health conditions, improved children’s health and nutritional status,^15,16^ reduced the prevalence of low birth weight and reduced the child mortality rate, both in general and due to poverty-associated causes.^6^ Information on the influence of the program on pregnant women’s food consumption and anthropometric patterns, however, is only just beginning to emerge.

Given the impact of the BFP on pregnant women’s health, our key hypothesis was that women who were beneficiaries of the BFP would have greater number of consultations in prenatal services, which would provide these women with a greater amount of nutritional counseling and monitoring from the healthcare teams. Therefore, women who were beneficiaries would achieve a positive impact on their nutrition and the beneficiaries would attain better control of weight gain during pregnancy. In addition, we postulated that the cash transfers would increase pregnant women’s food and nutritional security, through their adoption of healthier eating habits during pregnancy.

Thus, the BFP can be considered to be a social protection policy that contributes positively towards ensuring FNS. In addition, it is probable that the BFP can influence these relationships directly or indirectly, or in an intermediary fashion.

## OBJECTIVE

The aim of this study was to examine whether the Brazilian conditional cash transfer program was associated with changes to body mass index, food consumption and prenatal care, among pregnant women.

## METHODS

### Study design and sample

**Figure 1:**
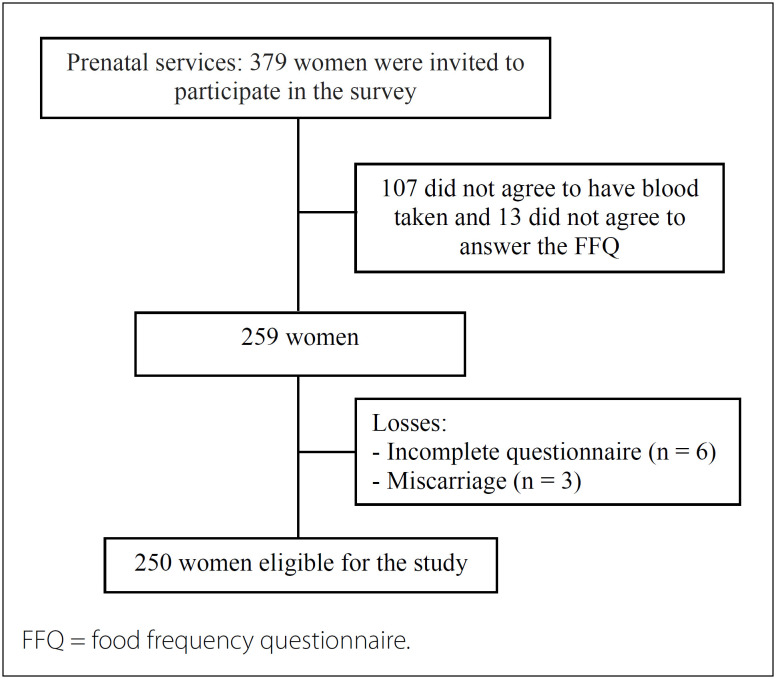
Flowchart of the design of the study conducted on pregnant women receiving prenatal services at a primary care unit in Santo Antônio de Jesus, Bahia, Brazil, 2017.

This was a prospective cohort study using a dynamic population of 250 pregnant women living in an urban area who were treated at Family Health Strategy (FHS) units in the city of Santo Antônio de Jesus, Bahia, in the northeastern region of Brazil. These women formed part of the NISAMI Cohort (Mother and Child Health Research Center; in Portuguese: Núcleo de Investigação em Saúde Materno-Infantil). The flowchart for the cohort is presented in Figure 1.

Pregnant women were recruited at prenatal services between August 2013 and December 2014. The monitoring lasted for nine months. When a pregnant woman was enrolled in the study after the first trimester of pregnancy, previous weight data were collected from the medical records available at the prenatal health unit. Data on socioeconomic, demographic, health and obstetric status and access to the BFP were collected at the time of the pregnant women’s enrollment in the study.

### Exclusion and inclusion criteria

The study included women who lived in the municipality’s urban area, were aged 18 years or over, had gestational ages of up to 34 weeks at the time of enrollment and were receiving prenatal care through the public healthcare system.

The criteria for exclusion after the baseline consisted of the following: twin/multiple pregnancy; adherence to a vegan diet; and renal, contagious, immunological and/or metabolic and HIV diseases confirmed via medical diagnosis. Absence of confirmation of gestational age through ultrasonography was also a reason for excluding pregnant women from the study. Gestational age was recorded from the first ultrasound, which was performed by the end of the first trimester and documented in the prenatal services.

Thus, 379 pregnant women met these criteria and were invited to participate in the study. They were invited to answer a closed-end questionnaire and to send blood samples to a clinical laboratory in the city after overnight fasting. Among these women, 107 refused to have blood collected and another 13 women refused to answer the food frequency questionnaire (FFQ). Consequently, a total of 259 pregnant women were eligible to participate. There were nine losses during the follow-up. Among these, six pregnant women did not fully complete the questionnaires and three had miscarriages. After these exclusions, 250 pregnant women were effectively included in the study and monitored for nine months (Figure 1).

### Bolsa Familia Program assessment

The Bolsa Familia Program (BFP) is a conditional cash transfer program that was created in October 2003 through Provisional Measure No. 132. It was aimed at poor and extremely poor families and used per capita family income as the inclusion criterion.^16^ The exposure variable consisted of receipt of BFP benefits.

### Outcome assessments

We adopted two continuous response variables: gestational body mass index (BMI) variation during gestation (first, second and third gestational trimesters); and dietary intake pattern relating to fatty acid, at the time of enrollment.

### BMI variation during pregnancy

For maternal weight measurements in the first, second and third gestational trimesters, we used a scale with a capacity of 150 kg and sensitivity of 100 g (Filizola, model 31 mechanical, Brazil). To measure height, a stadiometer was used, with a capacity of 2000 cm and sensitivity of 0.1 cm (Sanny, Brazil). Anthropometric measurements were made in duplicate. A maximum variation of 0.5 cm was accepted for length measurement, and a maximum variation of 100 g for weight.^17^

### Dietary intake assessments

To evaluate fatty acid consumption, the semi-quantitative FFQ was used.^18^ This instrument assesses 89 dietary sources of these lipids, with 13 possible responses for consumption, ranging from rarely/never to ≥ 3 times per day.

A photograph album of food portions and kitchen utensils was used to assist in making estimates of the portion sizes consumed, from the interviewees’ memory. Data on frequencies of consumption of foods and the portions consumed were inserted into a spreadsheet and calculated, using an adaptation from Santana et al.^19^

To analyze consumption, we used daily food consumption. Thus, all time intervals relating to polyunsaturated fatty acid consumption were converted into the daily frequency of polyunsaturated fatty acid consumption.^18^ In this, daily food intake frequency was assigned a value of one. For the weekly and monthly time intervals, the mean of the interval was divided by the period of fatty acid consumption: when weekly, it was assigned the value of 7, and when monthly, the value was 30.

The daily frequency of consumption of each food was used to form food groups that had the same nutritional characteristics, namely: milk and dairy products, fish, fruits and vegetables, olive oil, oilseeds and whole grains, refined grains, foodstuffs that were part of regional dishes, vegetable oil, sausages, salted meats and foods belonging to the snack and processed foods group.

### Statistical analyses

The power of this sample to detect an association between the Bolsa Familia Program and a pregnant woman’s nutritional status was 96%. This calculation was based on the prevalence of excess weight gain of 48.1%, among pregnant women in the municipality of the present study.^19^

Descriptive analyses were used to characterize the sample. Mean and standard deviation (SD) were used for the continuous variables (maternal age, number of years of education, family income, number of residents, number of gestational weeks, weight gain, prenatal visits and time when prenatal visits started). The sociodemographic and anthropometric characteristics of the pregnant women according to the BFP exposure variables were compared using Student’s t test.

In this study, the BFP was adopted as a categorical dependent variable, dichotomized into (0) beneficiaries of the program and (1) non-beneficiaries of the program. The main exposure variables were the fatty acid consumption pattern and gestational BMI, and these were used in a continuous form. The following covariates were considered in continuous format: maternal age, number of years of education, family income, number of residents, number of gestational weeks, BMI, body weight (BW), birthweight gain, prenatal visits and time when prenatal services started.

The BMI variable was constructed longitudinally, considering weight variation in the first, second and third gestational trimesters using generalized estimation equations. Later on, this variable in its continuous form was exported to the structural equation model (SEM) in order to evaluate the influence of the BFP on BMI variation during gestation. This strategy was adopted because the model in the SEM did not fit when the variable “gestational weight gain variation over time” was included in the confirmatory equation of the factorial analysis.

Dropout analyses were performed to investigate the presence of selection bias, through comparing the mothers who completed the study with those who were lost or excluded during the monitoring. The following variables were considered: age and consumption of mono and polyunsaturated fatty acids.

The baseline consumption of polyunsaturated fatty acids gave rise to two latent variables. These two variables are here denominated “Pattern 1” (milk and dairy products, fish, fruits and vegetables, olive oil, legumes, oilseeds and tubers and roots) and “Pattern 2” (refined grains, regional foods, vegetable oil, sausages, salted meats and snacks). These patterns were established internally in SEM by using confirmatory factorial analysis and were included in analyses in the continuous form. The composition of the fatty acid consumption pattern and its respective standardized coefficients are presented in Table 1.

**Table 1:** Sociodemographic and anthropometric characteristics of pregnant women who were beneficiaries and non-beneficiaries of the Bolsa Familia Program (BFP). Santo Antônio Jesus, Bahia, 2013 to 2014 (n = 250).

Characteristics (continuous variables)	BFP beneficiaries				P-value*
	YES		NO		
	Mean	SD	Mean	SD	
Maternal age in years	28.3	5.6	26.1	6.0	< 0.001
Number of years of education	9.9	2.8	10.8	2.9	< 0.001
Family income**	260.48	147.37	420.42	319.07	< 0.001
Number of residents in the home	4.0	1.0	3.0	1.0	0.002
Number of gestational weeks	17.4	7.1	16.3	6.6	0.002
BMI BW	25.8	5.7	23.8	4.2	< 0.001
Birthweight gain (kg)	12.9	4.9	12.8	4.9	0.705
Number of prenatal visits	6.7	2.1	7.3	2.0	0.999
Prenatal visits start (trimester)	2.8	1.6	2.3	1.6	< 0.001

* Student’s t test for independent samples

** United States dollars; BMI BW = pre-pregnancy body mass index.

The direct, indirect and total effects of the relationships studied were evaluated through standardized coefficients (SCs), and these were interpreted as a small effect (SC values close to 0.10 and -0.10), medium effect (SC values of 0.30 and -0.30) or strong effect (SC values > 0.50 and > -0.50).^20^ The RMSEA model (root of the mean square error of approximation) was used to evaluate goodness of fit.^21^ The statistical analyses were carried out using the Stata software (version 12.0; Stata Corporation, College Station, Texas, United States).

### Ethical statement

This study was approved by the Ethics Committee for Research involving Human Beings of the Universidade Federal do Recôncavo da Bahia (UFRB), under the number 241.225, dated April 9, 2013. All study procedures were carried out in accordance with the code of ethics of the World Medical Association (Declaration of Helsinki) for experiments involving humans. Informed consent was obtained for experimentation with human subjects and the privacy rights of human subjects were observed.

## RESULTS

### Description of participants

Out of the 259 pregnant women considered for the study, 250 women were included (Figure 1), and these participants contributed 750 observations on weight variation during monitoring. This loss of nine participants was recorded over nine months of follow-up and represented a loss rate of 3.47%. The results from the comparative analysis between the selected variables of mean age (P = 0.81), consumption of monounsaturated fatty acids (P = 0.80) and consumption of polyunsaturated fatty acids (P = 0.50) at baseline did not differ significantly between the women who completed the follow-up and the losses.

The sociodemographic and anthropometric characteristics of the pregnant beneficiaries (28.8%) and non-beneficiaries (71.2%) of the BFP are presented in Table 1. Women benefitting from the BFP were of greater age (28.3 years; SD = 5.6; P < 0.001), had had lower education (9.9 years; SD = 2.8; P < 0.001) and had lower family income (United States dollars, US$ 260.48; SD = 147.37; P < 0.001).

Information on the daily frequencies of consumption of food groups that were present in the diet of the pregnant beneficiaries and non-beneficiaries of the BFP is set out in Table 2. The pregnant beneficiaries of the BFP had basic food groups such as milk and dairy products, grains, legumes and oilseeds (i.e. components of the healthier pattern 1) in their daily consumption more frequently.

**Table 2:** Daily frequency of consumption of food groups in the diet of pregnant women who were beneficiaries and non-beneficiaries of the Bolsa Familia Program (BFP). Santo Antônio de Jesus, Bahia, 2013 to 2014 (n =250).

Food groups	BFP beneficiaries		Non-beneficiaries		P-value*
	Median freq	SD	Median freq	SD	
Milk and dairy products	2.0	1.16	2.0	1.29	0.174
Meat and eggs	1.17	0.72	1.17	0.77	0.524
Fish	0.19	0.24	0.16	0.24	0.119
Sausages	0.47	0.66	0.39	0.74	0.067
Salted meats	0.43	0.49	0.34	0.47	0.010
Innards (viscera)	0.14	0.21	0.08	0.24	0.001
Olive oil	0.30	0.41	0.33	0.55	0.183
Oil	1.09	0.38	1.05	0.37	0.095
Fat	1.5	1.13	1.38	1.02	0.055
Snacks	0.34	0.64	0.30	0.39	0.175
Regional foods	0.22	0.32	0.28	0.44	0.033
Grains	2.57	1.72	2.24	1.36	0.002
Whole grains	0.52	0.58	0.49	0.56	0.230
Legumes	1.12	0.75	1.01	0.61	0.016
Oilseeds	1.00	0.17	0.14	0.43	0.062
Fruits, vegetables and legumes	0.59	0.77	0.52	0.82	0.474

* Student’s t test for independent samples.

### Program influence on fatty acid dietary intake patterns and anthropometric outcomes

The composition of the fatty acid consumption pattern and its respective standardized coefficients are shown in Table 3. Pattern 1 consisted of the following food groups: milk and dairy products, fish, fruits and vegetables, olive oil, legumes, oilseeds and whole grains. The root and tuber food groups had the greatest contribution towards formation of this construct and the olive oil and fish groups contributed to a lesser extent. Pattern 2 consisted of refined grains, foodstuffs that were part of regional dishes, vegetable oil, sausages, salted meats and foods belonging to the snack and processed foods group. The food groups that contributed the most notably to this pattern were salted meats, processed foods and those that make up snacks.

**Table 3:** Structural equation modeling on the influence of the Bolsa Familia Program on dietary patterns. Santo Antônio de Jesus, Bahia, 2013 to 2014 (n = 250)

Effects		Standardized coefficient	P-value	95% CI
**DIETARY PATTERN 1** →				
	Milk and dairy products	0.34	< 0.001	0.26-0.43
	Fish	0.23	< 0.001	0.14-0.32
	Fruits and vegetables	0.30	< 0.001	0.22-0.59
	Olive oil	0.20	< 0.001	0.11-0.29
	Legumes	0.47	< 0.001	0.38-0.56
	Oilseeds	0.54	< 0.001	0.45-0.66
	Tubers and roots	0.67	< 0.001	0.58-0.75
**DIETARY PATTERN 2** →				
	Refined grains	0.27	< 0.001	0.18-0.36
	Regional foods	0.20	< 0.001	0.12-0.29
	Vegetable oil	0.23	< 0.001	0.15-0.32
	Sausages	0.40	< 0.001	0.33-0.49
	Salted meats	0.67	< 0.001	0.58-0.75
	Snacks	0.60	< 0.001	0.52-0.68
**Pattern 1** ← **Bolsa Familia Program**		-0.02	0.665	-0.11 – -0.075
**Pattern 2** ← **Bolsa Familia Program**		-0.10	0.034	-0.19 – -0.07
**BMI_T** ← **Bolsa Familia Program**		-0.12	0.001	-0.05 – -0.18
	Bolsa Familia Program ← age	-0.16	< 0.001	-0.10 – -0.23
	Bolsa Familia Program ← number of years of education	0.08	0.024	0.01-0.15
	Bolsa Familia Program ← income	0.22	< 0.001	0.14-0.28
	Bolsa Familia Program ← number of residents in the home	-0.05	0.097	-0.12 – -0.01
	Bolsa Familia Program ← parity	-0.005	0.09	-0.15 – -0.02
	Bolsa Familia Program ← prenatal care start	-0.10	0.004	-0.16 – -0.03
	Bolsa Familia Program ← number of prenatal care visits	0.08	0.017	0.01-0.14

Goodness-of-fit indicators for the model: root of the mean square error of approximation (RMSEA): 0.0001; n = 250: 750 observations; BMI_T: body mass index (BMI) in the three gestational trimesters.

The Bolsa Familia Program had a direct negative effect on consumption pattern 2 (SC = -0.10; P = 0.034) and on BMI during pregnancy (SC = -0.12; P = 0.001). This indicated that pregnant women who were beneficiaries of the program had lower adherence to pattern 2 fatty acid dietary intake (refined grains, *caruru, vatapá*, vegetable oil, sausages, salted meats and snacks) and presented lower BMI during the gestational cycle (Table 3).

Among the other variables evaluated, there were direct negative effects from age (SC = -0.16; P ≤ 0.001) and time when prenatal care started (SC = -0.10; P = 0.004) on the relationship between the BFP and anthropometric status and fatty acid dietary intake conditions throughout gestation, thus indicating that the beneficiaries were younger and began prenatal care earlier. In this relationship, the following variables showed positive effects: number of prenatal consultations (SC = 0.08; P = 0.017), family income (SC = 0.21; P = 0.001) and number of years of schooling (0.08; P = 0.024) showed positive effects. This indicated that the BFP had a positive impact on increased income, greater maternal education and greater number of prenatal consultations (Table 3).

## DISCUSSION

This study was one of the first in Brazil to investigate the influence of pregnant women’s participation in the Bolsa Familia Program on BMI during pregnancy. The results indicate that pregnant beneficiaries of the BFP had lower adherence to food consumption pattern 2, composed of foods and preparations containing high concentrations of fatty acids, vegetable oil, sausages, salted meats and salty snacks than non-beneficiary pregnant women.

In this context, the results suggest that, as a social policy, the Bolsa Familia Program exerts a protective effect on maternal nutritional health, through increasing access to and consumption of the traditional basic foodstuffs within a healthy diet among Brazilian families, consisting of milk and dairy products, beans, meat, eggs and grains. These food groups have an outstanding physiological function within the development of adequate gestation and maintenance of women’s weight throughout pregnancy. It is also worth noting that the results from this study can be interpreted in the light of proposals for social protection programs (PTCRs). These programs focus not only on direct cash transfers to families in order to alleviate poverty over the short term, but also on requirements that encourage beneficiaries to access healthcare and educational services.^6,16^

Population-based studies have shown that PTCRs improve the economic conditions of poor households, which in turn promotes greater access to food and contributes towards ensuring these households’ FNS.^15^ Accordingly, the results from this study substantiate the direct relationship between the protective aspect of cash transfer programs and the underprivileged population’s health and nutrition.

Among the direct effects of these programs are those relating to the gestational cycle, which ensure that pregnant women have access both to food and to prenatal and postpartum consultations, in addition to ensuring their participation in educational interventions relating to nutrition and health within the public healthcare network.^7,8,16^

In a qualitative and quantitative study carried out in municipalities of the state of Bahia, it was found that the Bolsa Familia Program was one of the programs within the National Food and Nutrition Policy that had the greatest coverage in the municipalities evaluated.^22^ There was greater monitoring of its requirements, specifically in the area of healthcare, in municipalities where the Family Health Strategy covered more than 70% of the population. This showed that dialogue between these two public health programs favored interaction between positive health and nutritional actions in the population.^22^

Thus, adherence to the population’s traditional diet and reduction of consumption of industrialized and processed foods, along with improvement of anthropometric conditions, may reflect the actions of the Bolsa Familia Program. These actions do not mitigate the social determinants but provide guidance that promotes adoption of health and nutritional practices that lead to a healthier lifestyle. The results from the Bolsa Familia Program are consistent with those from other studies conducted elsewhere in the world. In this regard, a randomized, cluster-controlled clinical trial found that both direct income interventions and direct food interventions improved the nutritional consumption and nutritional status of low-income pregnant women in Nepal, Asia. In addition, conditional cash transfers further improved food diversity in family units.^23^

However, a randomized clinical trial that was conducted to evaluate the effectiveness of cash transfers in relation to searching for higher-level infrastructure and equipment services, among low-income African pregnant women in Nairobi, Kenya, did not observe any positive impact on their search for quality services for childbirth delivery.^24^

Nonetheless, the results from a study on Brazilian families who were in situations of poverty corroborated previous results.^23^ Those families invested an average of US$ 61.61 more per year for purchasing food than did non-beneficiary families.^25^ Reports from beneficiaries have indicated that families increase their purchases of rice, beans,^26^ chickenmeat,^27^ grains, milk, sugar, cookies, meats, oils, eggs, fruits, roots and processed foods after being included in the Bolsa Familia Program.^14,26,27^ It was seen that the food groups most frequently purchased were sugars (78%), rice and cereals (76%) and milk (68%), with lesser acquisition of foods in the vegetable group (40%).

Research results from a region close to that of the present study showed that beneficiary families’ diets improved after the program had been established in that region, and that the money received was mainly spent on food (76.4%). There was also an increase in the variety of the foods consumed.^28^

Although the pregnant women of the present study included the Brazilian population’s basic healthy food groups in their daily diet (milk and dairy products, beans, meat, eggs and grains), low consumption of fruits and vegetables was registered. These results were similar to those of other investigations carried out among adult Bolsa Familia Program women, and indicated that there was greater access to food after they had been included in the Bolsa Familia Program. Nevertheless, their consumption of vegetables, fruits and legumes remained lower than expected.^29^

The results from the investigations have highlighted social, economic and cultural influences, as well as the geographical region’s dietary habits, in constructing the population’s dietary patterns. Accordingly, in less favorable socioeconomic contexts such as that of Brazil’s northeastern region, families have tended to adapt to subsistence mechanisms and buy the basic and cheaper foodstuffs that are part of the population’s diet, while those living in economically more developed regions have adopted greater consumption of processed foods.^26,27^

Thus, we have highlighted the monitoring and follow-up of pregnant Brazilian beneficiaries of the Bolsa Familia Program through the Family Health Strategy program, with regard to prenatal care actions, with more pronounced insertion in the northeastern region.^10^ This result may also be expressing a positive effect from interaction between the Bolsa Familia Program and the FHS program, with greater access to prenatal care for pregnant Bolsa Familia Program beneficiaries and implementation of nutritional and health monitoring interventions throughout the prenatal care period. The net result is a protective impact on maternal and fetal health.

Certainly, the focus of the Bolsa Familia Program has enabled increased family income, which is a major factor in expanding beneficiaries’ access to food. Thus, the beneficiaries’ average family income was slightly higher than the minimum wage at the time (US$ 260.48; SD = 147.37), although, as expected, below the income of non-beneficiaries (US$ 420.4; SD = 319.07).

The Bolsa Familia Program has also had impacts on the health of the maternal and child populations registered in epidemiological studies, which consisted of reducing the prevalence of low birthweight and the mortality rate among children under five years of age, both in general and due to poverty-related causes such as malnutrition and diarrhea.^6,30^ In addition, the program has had a positive influence on children’s nutritional status and health, as a result of greater monitoring and mother-child bonding, achieved through actions to prevent risk factors and promote a healthy lifestyle.^30^

Thus, it has been found that the Bolsa Familia Program has had a positive impact on the health of the population served, through contributing to reduction of malnutrition and FNI, increasing monthly family income and allowing access to food, for previously invisible vulnerable population groups.^28^ In this light, the Bolsa Familia Program stands out as an intersectoral strategy for social inclusion of vulnerable families, thereby promoting the human right to adequate food (HRAF) through the FNS guarantee.^11,31^

In this way, the decision of the United Nations Food and Agriculture Organization (FAO) to exclude Brazil from the hunger map in 2014, owing to an 82% reduction in the prevalence of malnutrition in the population, can be understood. In the FAO report, the Bolsa Familia Program was highlighted as one of the social interventions that was able to contribute results with social and human importance at a global level, given that it has played an important role in guaranteeing FNS and promoting HRAF among Brazilian families.^32^

### Strengths and limitations

This study had some limitations. For instance, information provided by Bolsa Familia Program beneficiaries was used. This may have led to information errors, since these women receiving the benefit may have felt uncomfortable about giving information or may have felt threatened by loss of the benefit if they talked about their condition. Moreover, the pregnant women’s level of physical activity was not analyzed.

Another limitation related to non-inclusion of rural pregnant women in the study sample. Inclusion of rural pregnant women in the sample should be considered in the future, since groups may have different access to healthcare services according to where they live. This can also occur with regard to food consumption. In studies in other countries, with different epidemiological contexts, it has been reported that pregnant women have differing health and nutrition vulnerabilities according to their area of residence.^33–35^ In our study, however, these differences may not have been significant, given that Family Health Strategy units that serve the population through health monitoring programs, including prenatal care, also exist in rural areas of the municipality. Regarding the losses during the follow-up, we can attest that these were small and were unlikely to have introduced any selection bias in the study.

The robust statistical analysis in our study was a strength. It can thus be concluded that studies that address the Bolsa Familia Program’s impact on pregnant women’s diet and nutrition are at an early stage. We also noted that there was a lack of study designs with robust methodology for dealing with primary data. From this perspective, the present investigation has contributed to filling the gap, through a longitudinal study on the influence of the Bolsa Familia Program on pregnant women’s diets and on maternal weight during pregnancy.

## CONCLUSION

This study showed the important role of health conditionalities in expanding access to prenatal services and nutritional guidance during pregnancy, as well as for monitoring and controlling weight gain during pregnancy. Accordingly, the improvement of pregnant women’s parameters may have resulted from the Bolsa Familia Program’s impact on families’ economic conditions, through increasing the population’s access to food staples and providing orientation sessions for improving the beneficiary population’s health and nutrition.

This study advances the understanding of the positive influence of a social protection policy on the food and nutrition of specific populations, such as the group of pregnant women in urban areas. However, further studies are needed, especially among pregnant women in rural areas, since these women may have different access to healthcare services.
